# Reverse shock index multiplied by Glasgow Coma Scale score (rSIG) is a simple measure with high discriminant ability for mortality risk in trauma patients: an analysis of the Japan Trauma Data Bank

**DOI:** 10.1186/s13054-018-2014-0

**Published:** 2018-04-11

**Authors:** Akio Kimura, Noriko Tanaka

**Affiliations:** 1 Department of Emergency and Critical Care, Center Hospital of the National Center for Global Health and Medicine, 1-21-1 Toyama, Shinjuku City, Tokyo, 162-8655 Japan; 2 Biostatistics Section, Department of Data Science, Clinical Science Center, National Center for Global Health and Medicine, 1-21-1 Toyama, Shinjuku City, Tokyo, 162-8655 Japan

**Keywords:** Systolic blood pressure, Heart rate, Glasgow Coma Scale score, In-hospital mortality, Japan Trauma Data Bank

## Abstract

**Background:**

The shock index (SI), defined as heart rate (HR) divided by systolic blood pressure (SBP), is reported to be a more sensitive marker of shock than traditional vital signs alone. In previous literature, use of the reverse shock index (rSI), taken as SBP divided by HR, is recommended instead of SI for hospital triage. Among traumatized patients aged > 55 years, SI multiplied by age (SIA) might provide better prediction of early post-injury mortality. Separately, the Glasgow Coma Scale (GCS) score has been shown to be a very strong predictor. When considering these points together, rSI multiplied by GCS score (rSIG) or rSIG divided by age (rSIG/A) could provide even better prediction of in-hospital mortality.

**Methods:**

This retrospective, multicenter study used data from 168,517 patients registered in the Japan Trauma Data Bank for the period 2006–2015. We calculated areas under receiver operating characteristic curves (AUROCs) to measure the discriminant ability by comparing those of SI (or rSI), SIA, rSIG, and rSIG/A for in-hospital mortality and for 24-h blood transfusion.

**Results:**

The highest ROC AUC (AUROC), 0.901(0.894–0.908) for in-hospital mortality in younger patients (aged < 55 years), was seen for rSIG. In older patients (aged ≥ 55 years), the AUROC of rSIG/A, 0.845(0.840–0.850), was highest for in-hospital mortality. However, the difference between rSIG and rSIG/A was slight and did not seem to be clinically important. rSIG also had the highest AUROC of 0.745 (0.741–749) for 24-h blood transfusion.

**Conclusions:**

rSIG ((SBP/HR) × GCS score) is easy to calculate without the need for additional information, charts or equipment, and can be a more reliable triage tool for identifying risk levels in trauma patients.

## Background

The shock index (SI), defined as heart rate (HR) divided by systolic blood pressure (SBP), should be more than 0.8–1.0 in patients with shock, with higher values indicating more severe shock than lower values [1, 2]. It is reported to be a more sensitive marker of shock than traditional vital signs alone [1–3] and a marker of significant injury when HR and SBP are normal, because SBP is a late marker of shock following trauma. SI is easily calculated at the bedside without the need for additional information or equipment, and it has been used to identify risk of mortality and the need for massive transfusion [4, 5], even in the presence of severe traumatic brain injury [6].

According to Zarzaur et al. [7, 8], SI is a better predictor of 48-h mortality than traditional vital signs. However, SI can underestimate the severity of underlying shock in older injured patients, because older patients tend to have higher baseline SBP even after injury. Among patients aged > 55 years, SI multiplied by age (SIA) may be a better predictor of early post-injury mortality than vital signs.

Separately, the Glasgow Come Scale (GCS) score [9, 10], which is utilized for consciousness level assessment at almost every emergency center worldwide, has been shown to be a stronger predictor of the probability of survival than SBP, respiratory rate (RR), age, and even injury severity [11, 12].

According to a research group in Taiwan [13, 14], the reverse (or inverse) shock index (rSI), defined as the ratio of SBP to HR, is preferable to shock index, because practitioners generally view unstable hemodynamic status as SBP lower than HR, not as HR higher than SBP as indicated by the SI. In other words, rSI should be < 1 in patients with shock, and the research group recommended using the concept that a higher rSI translates to a higher probability of survival. It may be worthwhile for to us to evaluate the superiority of rSI to SI.

On the other hand, using simple assessment tools, such as SI or rSI without needing to rely on hard-to-remember charts or equipment to identify injured patients at risk of early death, is of paramount importance to those caring for injured patients, especially in resource-constrained settings such as low-income and middle-income countries (LMICs), where millions of injury-related deaths annually occur.

### Objectives

Considering all of these points together, we hypothesized that SI divided by the GCS score (SI/G) might be a better predictor of post-injury mortality or of requirement for early blood transfusion and also that SIA divided by G (SIA/G) might provide even better prediction. Moreover, the reverse (or inverse) of these values—rSI multiplied by GCS score (rSIG) and rSIG divided by age (rSIG/A)—might be more suitable for clinical use. In this study, we examined these hypotheses using data from the Japan Trauma Data Bank (JTDB) [15].

## Methods

### Study design and data collection

This retrospective multicenter study used data from the JTDB, a nationwide trauma registry introduced in Japan in 2003 and which currently holds data from 256 hospitals. The JTDB was established by the Japanese Association for Trauma Surgery (Trauma Registry Committee) and the Japanese Association for Acute Medicine (Committee for Clinical Care Evaluation). Data are continuously recorded via the Internet and stored on a data server at the Association for Japan Trauma Care and Research (JTCR). The database contains patients’ demographic data (on age, sex, vital signs including GCS score on admission at emergency departments, mechanisms and types of injury, Abbreviated Injury Scale (AIS) codes recorded using AIS 90 Update 98, and the survival state at discharge from hospitals, etc.). Patients suspected to have an injury with AIS ≥3 are registered mainly from tertiary care, emergency centers. The annual report [15] summarizing the last 5 years of demographic data is available on the website of the JTCR.

**Fig. 1:**
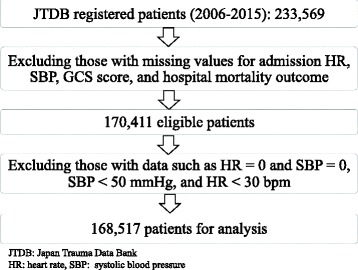
Patients flow chart. JTDB, Japan Trauma Data Bank; HR, heart rate, SBP; GCS, Glasgow Coma Scale

Data from 223,596 patients were obtained from the JTDB for the period 2006–2015. We excluded those patients with any missing values for HR, SBP, age, GCS score on admission, or hospital mortality. We excluded patients with data such as HR = 0 and SBP = 0, because calculations of SI or rSIG became infinite. We also excluded patients with very low blood pressure <50 mmHg and with very low heart rate <30 beats per minute (bpm), because the data were unrealistic and unreliable, and because SI and rSIG became extremely high or extremely low (Fig. 1).

### Measurements

SI was calculated in all eligible patients by dividing HR (in bpm) on admission by the SBP on admission measured in millimeters of mercury (mmHg). In accordance with the literature, we calculated simplified regression models (SRM) for probability of survival (logit = coded GCS + coded SBP + coded age – constant) [12] and GCS, age, and SBP (GAP) score [16], which were used to compare the performance of survival prediction. The GAP scoring system is a summation of the GCS point (from 3 to 15 points), age points (<60 years, 3 points), and SBP points (>120 mmHg, 6 points; 60–120 mmHg, 4 points). The score ranges from 3 to 24 with lower values indicating greater risk of in-hospital death.

### Statistical methods

Receiver operator characteristic curves (ROCs) were generated to illustrate the impact of shifting the positive cutoff value on true-positive (sensitivity) and false-positive (1 − specificity) rates. The area under the ROC curve (AUROC) was calculated to measure the ability of SI, SIA, SI/G (rSIG), and SIA/G (rSIG/A) to predict hospital mortality outcomes and 24-h blood transfusion outcomes. AUROCs were compared using the technique described by DeLong et al. [17]. JMP version 11 and JMP pro Version 13 software (SAS Institute, Cary, NC, USA) were used for all statistical analyses.

## Results

### Baseline data

Because of a large number of missing values, the number of the eligible patients became 170,411. Excluding patients with data such as HR = 0, SBP = 0, SBP <50 mmHg, and HR <30 bpm, the analyzed population comprised 168,517 patients (Fig. 1). Demographics of the analyzed patients and the eligible patients are shown in Table 1. In spite of those exclusions, the distributions of demographic data were also the same.

**Table 1:** Patient demographics

	Analyzed patients	Eligible patients
Patients (number)	168,517	170,411
Age (years)	56 (8–92)	56 (8–92)
Male patients	63.6%	63.6%
Vital signs on admission		
Heart rate (bpm)	87 (55–135)	87 (55–136)
Systolic blood pressure (mmHg)	137 (78–203)	136 (70–203)
Glasgow Coma Scale score	13 (3–15)	13 (3–15)
Blunt trauma	92.3%	92.3%
Injury Severity Score	15 (9–41)	15 (9–43)
24-h blood transfusion	13. 5%	13. 8%
In-hospital mortality	6.4%	6.4%
	Mean (95% CI)	

Among the patients analyzed, falls were the most frequent mechanism of injury (44.5%; falls on the ground 23.7%, from stairs 11.8%, and from a height 9.0%), followed by a motorcycle crash (15.7%), motor vehicle crash (11.4%), pedal cyclist accident (7.9%), pedestrian accident (7.4%), and other (13.1%).

### Outcomes

**Fig. 2:**
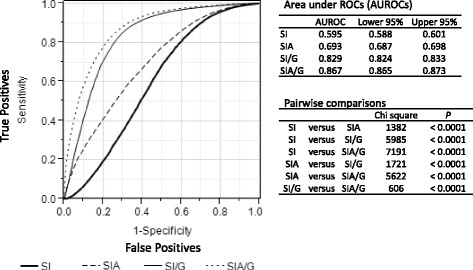
Comparisons of receiver operating characteristic (ROC) curves for in-hospital mortality. Bold line, shock index (SI); dashed line, SI × age (SIA); solid line, SI ÷ Glasgow Coma Scale (GCS) score (SI/G); dotted line, SIA ÷ GCS score (SIA/G)

ROC curves for in-hospital mortality with SI, SIA, SI/G, and SIA/G are shown in Fig. 2. Statistical differences (*p* < 0.0001) were seen among the AUROCs. The AUROCs were markedly higher for the SI/G and SIA/G measures, with much better discriminant ability for prediction of in-hospital mortality.

**Fig. 3:**
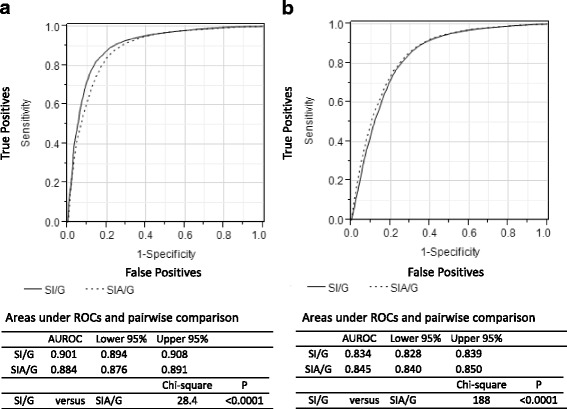
Comparison of receiver operating characteristic (ROC) curves for in-hospital mortality by age. Solid line, shock index ÷ Glasgow Coma Scale score (SI/G); dotted line, shock index × age ÷ Glasgow Coma Scale score (SIA/G). **a** ROC curves in younger patients (aged < 55 years). **b** ROC curves in older patients (aged ≥ 55 years). AUROC, area under the ROC

As in the literature [7, 8], we compared SI/G and SIA/G between younger patients (< 55 years) and older patients (≥ 55 years): which showed the AUROC for SI/G was higher in younger patients, AUROC 0.901 (0.894–0.908) (*p* < 0.0001), and the AUROC for SIA/G was higher in older patients, AUROC 0.845 (0.840–0.850) (*p* < 0.0001) (Fig. 3). SI/G seemed to be a slightly better predictor of in-hospital mortality among younger patients, while SIA/G seemed slightly better among older patients.

Mean, median, and standard deviation values for the calculated items were 0.7, 0.6, and 0.3 for SI; 0.06, 0.44, and 0.06 for SI/G; 3.2, 2.4, and 3.6 for SIA/G; 1.7, 1.6, and 0.5 for rSI; 23, 23, and 9.0 for rSIG; and 0.6, 0.4, and 0.7 for rSIG/A, respectively. The means and medians of the distributions of the reverse (or inverse) values were closer together than those of SI, SI/G, and SIA/G themselves, meaning that the reverse (or inverse) values deviated less from the Gaussian distribution. Using rSIG or possibly rSIG/A seems to be easier and appears to facilitate the identification of clear cutoffs. The ROC curve of SI/G is the same as that of rSIG, because the latter is just the inverse value of the former. That of SIA/G is also the same as that of rSIG/A.

**Fig. 4:**
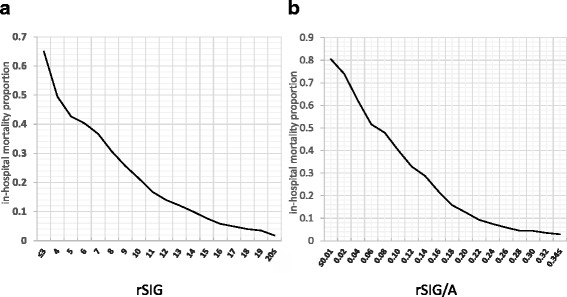
Proportion of patients with in-hospital mortality by reverse shock index × Glasgow Coma Scale score (rSIG) and by rSIG ÷ age (rSIG/A). The y axis shows the outcome variable of the proportion of patients with in-hospital mortality. The x axis shows rSIG (**a**) or rSIG/A (**b**)

Figure 4 shows graphs of in-hospital mortality by rSIG (Fig. 4a) and by rSIG/A (Fig. 4b). The lines representing the proportion with in-hospital mortality show a gradual decrease with increases in rSIG and rSIG/A.

There were statistical differences (*p* < 0.0001) among the AUROCs for the 24-h blood transfusion outcome according to SI (0.701(0.697–0.705)), rSIG (0.745(0.741–749)), and rSIG/A (0.731(0.727–0.735)). Although the AUROC was highest for rSIG, the difference was relatively small between rSIG and rSIG/A.

**Fig. 5:**
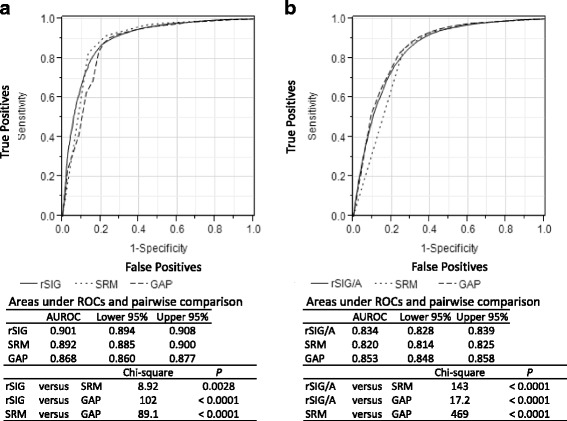
Comparisons of receiver operating characteristic (ROC) curves for in-hospital mortality. The vertical axis shows sensitivity, and the horizontal axis shows 1-specificity. **a** ROCcurves in younger patients (aged < 55 years). Solid line, reverse shock index × Glasgow Coma Scale score (rSIG); dotted line, simplified regression model (SRM); dashed line, Glasgow Coma Scale, Age, and systolic blood pressure (GAP) score. **b** ROC curves in older patients (aged ≥ 55 years). Solid line, rSIG ÷ age (rSIG/A); dotted line, SRM; dashed line, GAP score

A comparison of the AUROCs for SRM, GAP, rSIG, and rSIG/A is shown in Fig. 5. Among younger patients, the AUROC for rSIG was slightly higher (*p* = 0.0028) than that for SRM and was higher (*p* < 0.0001) than that for the GAP score (Fig. 4a). The AUROC for rSIG/A was higher (*p* < 0.0001) than that for SRM, but was lower (*p* < 0.0001) than that for GAP in older patients (Fig. 4b).

## Discussion

Injuries are a growing public health concern, resulting in the death of about 5 million people annually worldwide [18]. The majority of injury-related deaths occur in LMICs, where human and technological resources for trauma care are limited. Identification of injured patients at risk of early death or those at very low risk of mortality is crucial to those providing triage at overcrowded emergency centers or after mass casualty incidents. Moreover, quality improvement (QI) in trauma care systems is an important component in strengthening health care systems in LMICs, and objective comparison of outcomes with risk adjustment is essential for QI [19]. Various methods have been developed for this purpose. The Injury Severity Score (ISS) [20] consists of Abbreviated Injury Scale [21] codes for the three most severely injured body regions. The Revised Trauma Score (RTS) [22] is a physiological score consisting of GCS score, RR, and SBP. The Trauma and Injury Severity Score (TRISS) [23] is the most well-known logistic regression model, which predicts survival probabilities (Ps) and comprises the ISS, RTS, age, and mechanism of injury. However, these methods are cumbersome and impractical, especially in LMICs, due to difficulties in information collection and real-time calculation in the absence of charts or computers. Many simplifications [24, 25] have been proposed around the world, but these still require complicated calculations.

Over the last 10 years, we have developed simplified logistic regression models using minimum predictors [12, 26] that are obtainable even in LMICs. If a slight reduction in accuracy from the TRISS method is permitted, the simplest regression model for Ps that we have developed so far is (coded GCS + coded SBP + coded age – constant) [12]. This model was developed by simplification of the coefficients of a logistic regression model for survival using the three coded predictors of GCS (0–4), SBP (0–4), and age (0 or 1), which are the same as the coded values used for the RTS. However, a coding chart for GCS, SBP, and age is still required. Another Japanese research group developed the GAP score (3–24) [16] as a scoring system for severity using GCS score, age, and SBP on admission as predictors. Although the GAP score seems to strongly predict in-hospital mortality, it is almost impossible to determine without a scoring chart.

SI was first described by Allgower and Burri in 1967 [27], and has been reported as a more sensitive marker of shock and of the likelihood of success of resuscitation efforts than traditional vital signs alone [1–3]. SI is easily calculated at the bedside without the need for additional information or equipment, and it has also been used to identify mortality and the need for massive transfusion [4, 5] even in the presence of severe traumatic brain injury. SI is thus practicable in LMICs, not only for outcome prediction, but also for real-time assessment of trauma triage at emergency centers. However, it should be noted that SI may underestimate the severity of underlying shock in injured patients who are older because they tend to have higher baseline SBP even after injury. According to Zarzaur et al. [7, 8], among patients aged > 55 years, SIA may be a better predictor of early post-injury mortality than vital signs.

Meanwhile, the globally recognized GCS score has been utilized by virtually every physician who has managed emergency patients, and has proven to be a very strong predictor [11, 12]. As mentioned earlier, these points led us to explore whether SI divided by GCS score (SI/G) is a better predictor of in-hospital mortality or of requirement for early blood transfusion and whether SIA divided by G (SIA/G) is better in older patients. Moreover, distribution of the reverse (or inverse) of these values, namely rSIG and rSIG/A, has less deviation from the Gaussian distribution, and provides easier figures for clinical use.

To the best of our knowledge, this is the first report to show that rSIG and rSIG/A are excellent tools for identifying high-risk trauma patients. Like SI or SIA, rSIG on admission is very easy to calculate in emergency departments without the need for additional imaging, blood tests, or hard-to-remember coding systems. In addition, among the calculated values used in this study the AUROC was highest for rSIG for predicting survival in younger patients (< 55 years). A higher rSIG means better survival (or lower in-hospital mortality) (Fig. 3a). Among older patients (≥ 55 years), as with rSIG, a higher rSIG/A indicates better survival (or lower in-hospital mortality) (Fig. 3b). However, these statistical differences do not seem to have clinical relevance. Moreover, in our results, rSIG seemed to be a better predictor of the need for early (24-h) blood transfusion. Thus, it is easier and sufficient to use only rSIG in emergency medical settings.

In addition to being easy to calculate without scoring, the AUROC for rSIG was slightly larger than that for SRM and was larger than that for the GAP score in younger patients (Fig. 5a). The AUROC for rSIG/A was larger than that for SRM, but lower than that for GAP in older patients. However, these statistical differences seem to be clinically unimportant. These findings indicate that rSIG and rSIG/A have discriminant ability for in-hospital mortality risk as good as previously developed prediction methods that used only vital signs and age. This indicates that rSIG and rSIG/A can be utilized as a simple tool for risk adjustment in LMICs. Considering it is easier to calculate, again using only rSIG may be more realistic and satisfactory.

Furthermore, because these methods offer the advantage of rapid, real-time calculation in emergency centers, especially for rSIG, it can be used as a second triage tool for traumatized victims during mass causality incidents. The values of rSIG can enable rapid sorting of severely injured patients at the entrance on arrival.

### Limitations

Our study has some limitations that might be potential sources of bias. First, this was a retrospective study, and so prospective corroboration will be needed for validation. Second, the results were confirmed using only Japanese data, and thus international validation, especially in LMICs, will be required for worldwide use in the future. Cutoff values may differ among countries and may reflect the quality of the trauma system. Third, in the JTDB, 92.3% of cases involve civilian blunt trauma, so the results of this study may not be applicable to patients with penetrating injuries, particularly those with firearm injuries.

## Conclusion

Despite these limitations, our results indicate that the rSIG ((SBP/HR) × GCS score) is easy to calculate without the need for additional information, hard-to-remember charts, or equipment, and it is a sensitive negative predictor for high-risk trauma patients. A higher rSIG means lower in-hospital mortality. These are practicable measures for real-time assessment and sorting traumatized patients by risk in overcrowded emergency centers, and might have more value in resource-constrained settings such as LMICs.

## Abbreviations

AIS: Abbreviated Injury Scale
AUROC: Area under the receiver operator characteristic curve
bpm: Beats per minute
GAP: Glasgow Coma Scale score, age, and systolic blood pressure
HR: Heart rate
ISS: Injury severity score
JTCR: Japan Trauma Care and Research
JTDB: Japan Trauma Data Bank
LMICs: Low-income and middle-income countries
ROC: Receiver operator characteristic curve
RR: Respiratory rate
rSI: Reverse shock index
rSIG/A: Reverse shock index divided by age
RTS: Revised Trauma Score
SBP: systolic blood pressure
SI: Shock index
SIA: Shock index multiplied by age
SRM: Simplified regression models
TRISS: Trauma and Injury Severity Score
